# Sequence Search Algorithms for Single Pass Sequence Identification: Does One Size Fit All?

**DOI:** 10.1002/cfg.61

**Published:** 2001-02

**Authors:** K. Cara Woodwark, Simon J. Hubbard, Stephen G. Oliver

**Affiliations:** 1 Department of Biomolecular Sciences UMIST, PO Box 88 Manchester M60 1QD UK; 2 School of Biological Sciences University of Manchester Oxford Road Manchester M13 9PT UK

## Abstract

Bioinformatic tools have become essential to biologists in their quest to understand the vast quantities of sequence data, and now whole genomes, which are being produced at an ever increasing rate. Much of these sequence data are single-pass sequences, such as sample sequences from organisms closely related to other organisms of interest which have already been sequenced, or cDNAs or expressed sequence tags (ESTs). These single-pass sequences often contain errors, including frameshifts, which complicate the identification of homologues, especially at the protein level. Therefore, sequence searches with this type of data are often performed at the nucleotide level. The most commonly used sequence search algorithms for the identification of homologues are Washington University’s and the National Center for Biotechnology Information's (NCBI) versions of the BLAST suites of tools, which are to be found on websites all over the world. The work reported here examines the use of these tools for comparing sample sequence datasets to a known genome. It shows that care must be taken when choosing the parameters to use with the BLAST algorithms. NCBI’s version of gapped BLASTn gives much shorter, and sometimes different, top alignments to those found using Washington University’s version of BLASTn (which also allows for gaps), when both are used with their default parameters. Most of the differences in performance were found to be due to the choices of default parameters rather than underlying differences between the two algorithms. Washington University’s version, used with defaults, compares very favourably with the results obtained using the accurate but computationally intensive Smith–Waterman algorithm.


**Comparative and Functional Genomics**
*Comp Funct Genom* 2001; **2**: 4–9.

**Short Communication**

### Sequence search algorithms for single pass sequence identification: does one size fit all?

K. Cara Woodwark[1]*, Simon J. Hubbard[1] and Stephen G. Oliver[2]
[1] *Department of Biomolecular Sciences, UMIST, PO Box 88, Manchester M60 1QD, UK*
[2] *School of Biological Sciences, University of Manchester, Oxford Road, Manchester M13 9PT, UK*

**Correspondence to: K. Cara Woodwark, Department of Biomolecular Sciences, PO Box 88, Manchester M60 1QD, UK.*

### Abstract

**Bioinformatic tools have become essential to biologists in their quest to understand the vast quantities of sequence data, and now whole genomes, which are being produced at an ever increasing rate. Much of these sequence data are single-pass sequences, such as sample sequences from organisms closely related to other organisms of interest which have already been sequenced, or cDNAs or expressed sequence tags (ESTs). These single-pass sequences often contain errors, including frameshifts, which complicate the identification of homologues, especially at the protein level. Therefore, sequence searches with this type of data are often performed at the nucleotide level. The most commonly used sequence search algorithms for the identification of homologues are Washington University's and the National Center for Biotechnology Information's (NCBI) versions of the BLAST suites of tools, which are to be found on websites all over the world. The work reported here examines the use of these tools for comparing sample sequence datasets to a known genome. It shows that care must be taken when choosing the parameters to use with the BLAST algorithms. NCBI's version of gapped BLASTn gives much shorter, and sometimes different, top alignments to those found using Washington University's version of BLASTn (which also allows for gaps), when both are used with their default parameters. Most of the differences in performance were found to be due to the choices of default parameters rather than underlying differences between the two algorithms. Washington University's version, used with defaults, compares very favourably with the results obtained using the accurate but computationally intensive Smith–Waterman algorithm. Copyright © 2001 John Wiley & Sons, Ltd.**

**Keywords: BLASTn; sequence search algorithm; EST; cDNA; yeast; single pass sequence**

### Introduction

Sequence search algorithms are the keystone of bioinformatic tools, the most popular of which is probably the Basic Local Alignment Search Tool (BLAST; Altschul *et al.*, 1990) algorithm. When a biologist obtains a new sequence, one of the first tasks he/she undertakes is to 'BLAST' it against a database of choice in order to check the sequence or to try to discover more about it. If this sequence is 'single pass' (sequenced only once), such as an EST or a sample shotgun sequence, then it may contain undetected sequencing errors, such as single nucleotide insertions or deletions. These frameshift errors naturally cause problems when the sequence is translated into protein. Alternatively, the sequence of interest may not code for protein; for example, when upstream regions are compared to elucidate promoter regions or the sequence codes for rRNA or tRNA molecules. In these instances, nucleotide sequences are often compared to sequences from closely related species, most commonly by using BLASTn. These types of sequence data are now being produced at a phenomenal rate, and so this sequence 'identification' is often automated. If automated, the default BLAST parameters tend to be used, as they are often optimized to give the best results with a range of sequences, as well as allowing for consistency of results between runs.

Copyright © 2001 John Wiley & Sons, Ltd.

Single sequences are also usually analysed with BLAST using the default parameters, as when web-based forms are used they often do not allow for BLASTn parameters to be changed. This is due to the problems in calculating the sum statistics for BLAST when gaps are allowed in the alignments. When run with anything other than the default parameters, the Washington University version of BLASTn displays the following:

> 'WARNING:
>
> Precomputed values for Lambda, K, and H are unavailable for the +1, −3 scoring matrix, when used with gap penalties of −5 and −2. Unless overridden on the command line, the values computed for ungapped alignments will be used instead, but may yield P-values that are unduly low.'

There are two versions of BLAST which allow gaps, available on the web (or for download): from Washington University (wuBLAST) (Altschul *et al.*, 1990) and from the National Centre for Biotechnology Information (ncbiBLAST) (Altschul *et al.*, 1997). These vary in the default parameters available for BLASTn (see Table 1) as well as how the algorithms introduce gaps into the alignment.

The *Saccharomyces cerevisiae* genome sequence has been available for some time (Goffeau *et al.*, 1996) but there are still many gaps in our knowledge of its genes and of its relationship with other members of the genus *Saccharomyces*. In order to try and fill in some of these gaps, we are performing sample shotgun sequencing on the genomes of other members of the genus. The sequences used for the comparisons described here are from the *Saccharomyces sensu stricto* yeast *S. bayanus*, which is closely related to *S. cerevisiae* (Ryu *et al.*, 1996; Naumov 1987; Fischer *et al.*, 2000). This work was the first step in identifying gene homologues between *S. bayanus* and *S. cerevisiae.* To this end, coding regions in *S. bayanus* were identified by comparing the sample sequences against a database of *S. cerevisiae* coding regions (obtained from the KEGG ftp site **ftp://kegg.genome.ad.jp/pub/genomes/sequences/S.cerevisiae**).

### Materials and methods

### Sequence search algorithms

Washington University's Blast version 2.0a19MP, available from **http://blast.wustl.edu/,** was used with default parameters as well as with the default parameters of ncbiBLAST, as detailed in Table 1.

National Center for Biotechnology Information's gapped Blast version 2.0.9 (Altschul *et al.*, 1997) (available from **http://www.ncbi.nlm.nih.gov/BLAST**) was used with default parameters as well as with the default parameters of wuBLAST, as shown in Table 1.

Fasta 3 (Pearson and Lipman, 1988) and the SSearch implementation of Smith–Waterman (Smith and Waterman, 1981) (available from **http://www.ebi.ac.uk/FTP/**) came from the same

Table 1. Parameters used in the different BLAST comparisons

| **Algorithm** | **Washington Uni's BLASTn** | | **ncbiBLASTn** | |
|---|---|---|---|---|
| Origin of parameters | wuBLASTn (wuBLASTn) | ncbiBLASTn (wu_ncbiPar) | ncbiBLASTn (ncbiBLASTn) | wuBLASTn (ncbi_wuPar) |
| Match | 5 | 1 | 1 | 5 |
| Mismatch | −4 | −3 | −3 | −4 |
| Gap opening penalty | −10 | −5 | −5 | −10 |
| Gap extension penalty | −10 | −2 | −2 | −10 |
| Filter | False | True | True | False |

```
GATCGATCGATGCT
|||||||||||||| 
GATTG---GATGCT
1  2 3 4
```

1. Match—positive score.
2. Mismatch—negative score/penalty.
3. Gap opening—this penalty applies per gap.
4. Gap extension—a gap extension penalty is added for each missing nucleotide in the gap.

Copyright © 2001 John Wiley & Sons, Ltd. 

suite of tools. These were also used with their default parameters (see Table 3).

All sequence search algorithms were run on two processors of a four-processor SGI Origin and an SGI $O_2$.

### Data

The *S. cerevisiae* DNA protein-coding database was taken from Kegg (Kyoto Encyclopaedia of Genes and Genomes) ftp site (**ftp://kegg.genome.ad.jp/pub/genomes/sequences/S.cerevisiae**) The *S. bayanus* sequences were from shotgun-cloned sample sequences, sequenced by the Washington University Sequencing Centre, and kindly made available by Mark Johnston. There are 909 sequences with an average length of 403 nucleotides.

### Results

In a trial of sample sequence identification, using the two versions of BLASTn (default parameters), it was discovered that they found not only different lengths and numbers of database matches (hits) but also different hits and even different top hits.

The graph in Figure 1 shows a comparison of the number of hits found for wuBLASTn and ncbiBLASTn for a sample of 909 sample shotgun sequences from *S. bayanus*, 'BLASTed' against the DNA database of *S. cerevisiae* coding regions. These species are closely related, so that homologue identification should be possible at the DNA-sequence level. As may be seen from Figure 1, ncbiBLASTn (mean = 16.1) normally finds more hits than wuBLASTn (mean = 5.5). However, there are a number of sequences for which wuBLASTn finds a greater number of hits as, by default, filtering for simple sequences is not switched on in wuBLASTn.

Table 3. Default parameters for the sequence search algorithms

| Parameters | wuBLASTn | ncbiBLASTn | Smith–Waterman | Fasta |
|---|---|---|---|---|
| Match | 5 | 1 | 5 | 5 |
| Mismatch | −4 | −3 | −4 | −4 |
| Gap opening penalty | −10 | −5 | −16 | −16 |
| Gap extension penalty | −10 | −2 | −4 | −4 |

Figure 2 shows a comparison of alignment lengths for those alignments that were found in common between the two versions of BLASTn for the same query sequence. In all cases, wuBLASTn (mean = 251.71 bp) finds alignments of the same length or longer than ncbiBLASTn (mean = 136.12 bp). The average length of the query sequences is only 403 nt and many sequences are only partially coding, if at all. These results were unexpected and invited a more detailed analysis.

Using the same set of sequences from *S. bayanus*, the two BLAST2 versions were compared again but, this time, their parameters were changed to the default values of the other algorithm. Therefore, wuBLASTn was used with NCBI's parameters (wu_ncbiPar) and ncbiBLASTn with those of wuBLASTn's parameters (ncbi_wuPar), as shown in Table 1. The results of this comparison are shown in Table 2.

The 'hits in common', as shown in Table 2, were calculated between the two versions of BLASTn with their original parameters and between the two versions with exchanged parameters. Figure 3 shows that wu_ncbiParBLASTn (i.e. wuBLASTn with NCBI's default parameters) usually finds more hits than ncbi_wuParBLASTn. Figure 4 shows that

Table 2. Summary of the effects of exchanging BLASTn parameters between ncbiBLASTn and WuBLASTn

| Algorithm | Washington Uni's BLASTn | | ncbiBLASTn | |
|---|---|---|---|---|
| Origin of parameters | wuBLASTn wuBLASTn | ncbiBLASTn wu_ncbiPar | ncbiBLASTn ncbiBLASTn | wuBLASTn ncbi_wuPar |
| No. of hits (range) | 0–259 | 0–125 | 0–128 | 0–234 |
| Mean no. of hits | 5.5 | 11.9 | 16.1 | 11.6 |
| Total no. of hits | 5365 | 10794 | 14644 | 10592 |
| Total no. of hits in common (as % of total) | 1517 (28.27%) | 2475 (22.93%) | 1517 (10.35%) | 2475 (23.37%) |
| **For the hits found in common** | | | | |
| Alignment length (range) | 28–752 | 13–540 | 14–540 | 15–752 |
| Mean length | 251.7 | 75.5 | 136.1 | 148.0 |

Copyright © 2001 John Wiley & Sons, Ltd.

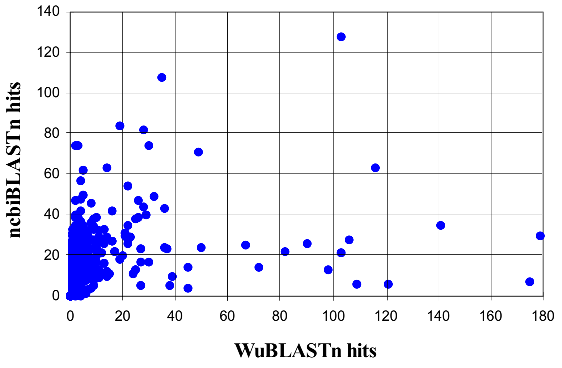

**Figure 1.** Comparison of the number of hits found for the same query sequence, by wuBLASTn and ncbiBLASTn

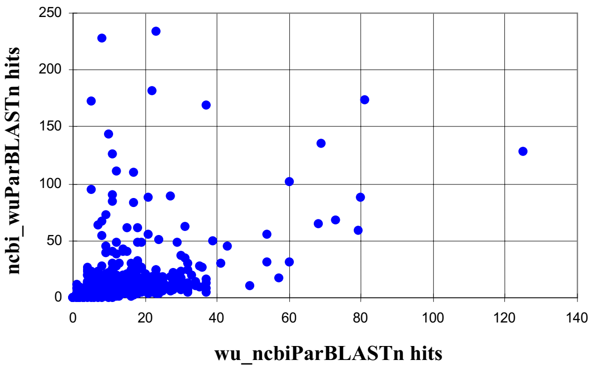

**Figure 3.** Number of hits found, for ncbi_wuParBLASTn and wu_ncbiParBLASTn

NCBI (with wuBLASTn's default parameters, i.e. ncbi_wuPar) finds the longer alignments. However, it is not a complete role reversal, as may be seen from Table 2. The mean number of hits for wu_ncbiParBLASTn is lower than for ncbiBLASTn, even though they are using mostly the same parameters. Also, the mean number of hits for ncbi_wuParBLASTn is still much higher than for wuBLASTn. The same story is repeated for average alignment lengths. Therefore, not all the differences are due to the matrices and gap penalties used. Other underlying differences between the two versions of BLASTn must be responsible, but the matrix and gap penalties play a very important role.

These comparisons show that the database sequence matches found by the two versions of BLASTn were different, but not which algorithm is better at actually detecting homologues at the nucleotide level. To find out which of the two BLAST versions found the 'right' hits, they were both compared to FASTA and the 'gold standard' of sequence search algorithms—Smith–Waterman, using default parameters again (see Table 3). The results of this second comparison are summarized in Tables 4 and 5.

The same top 'hit' is found by all four methods 536 times for the 909 sample sequences. It should be noted that not all of these sample sequences are coding, so that not all sequences will find matches to the *S. cerevisiae* coding regions. Not surprisingly, FASTA and Smith–Waterman, since they are based on the same algorithm and use almost the same default parameters, find the most hits in common. wuBLASTn and Smith–Waterman have the next most in common, with ncbiBLASTn finding far fewer hits in common with Smith–Waterman. This pattern is repeated with the alignment lengths, although wuBLASTn actually produces, on average, slightly longer alignments than FASTA.

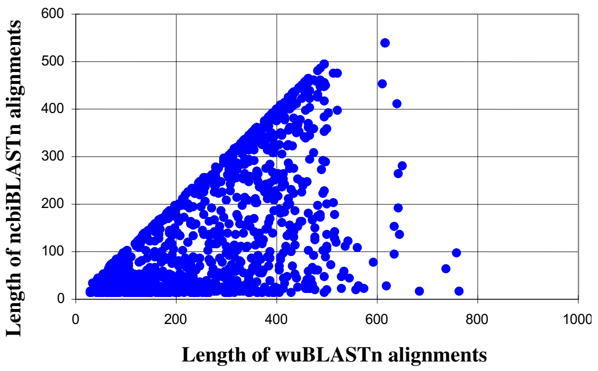

**Figure 2.** Comparison of alignment lengths for hits in common between wuBLASTn and ncbiBLASTn

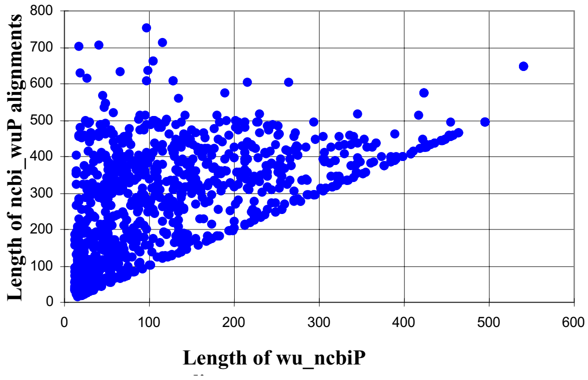

**Figure 4.** Length of alignments of hits found in common for ncbi_wuParBLASTn and wu_ncbiParBLASTn

Copyright © 2001 John Wiley & Sons, Ltd. 

Table 4. Comparison of top alignments found for the sequence search algorithms as compared to Smith–Waterman

| | Top hits in common (out of 909) |
|---|---|
| All found no hits | 0 |
| All found same top hits | 536 |
| FASTA = Smith–Waterman | 677 |
| WuBLASTn = Smith–Waterman | 641 |
| ncbiBLASTn = Smith–Waterman | 556 |
| WuBLASTn = ncbiBLASTn | 557 |

NcbiBLASTn's alignments are very much shorter (see Table 5).

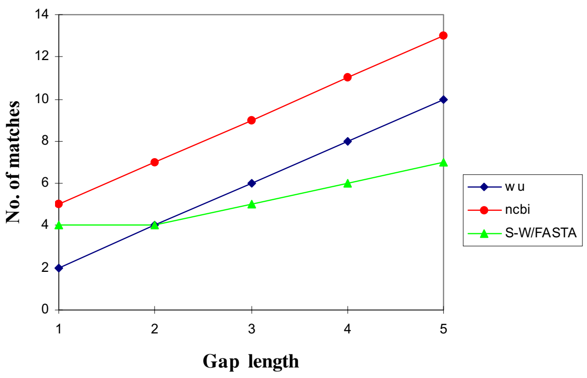

**Figure 5.** Number of nucleotide matches needed to allow for a gap in an alignment

### Discussion

The fact that ncbiBLASTn finds different top 'hits' and shorter alignments to the other algorithms is probably due to the matrix and gap penalties used, which do not allow for the gaps needed to extend alignments for this type of data. As may be seen from Table 3, wuBLASTn, FASTA and Smith–Waterman all use the same match/mismatch scores, although their gap penalties differ. Therefore, all three need fewer matches than ncbiBLASTn to allow for the insertion of a gap, as may be seen in Figure 5. Of course, the ability to extend alignments by inclusion of gaps and mismatches depends on the value of X, the extension threshold (Altschul *et al.*, 1990). However, the opportunity to change this parameter is rarely given on web-based BLAST servers. WuBLASTn's non-affine gap penalties mean that the programme tends to open gaps but not extend them, so that it will include short gaps, but not long ones. This allows for the frameshift errors found in single-pass sequence data, but not for long insertions or deletions.

It, has been suggested by Wolfe and co-workers (Wolfe and Shields, 1997; Keogh *et al.*, 1998; Seoighe and Wolfe, 1999) that the *Saccharomyces sensu stricto* species have undergone a complete genome duplication during the course of their evolution. If this is so then it is not possible to know whether the homologue or the paralogue of a particular sample sequence has been found (this is because there may be differences in the pattern of gene loss during the evolution of the two species). Therefore, comparing WuBLASTn and ncbiBLASTn's 'top hits' with Smith–Waterman does not give us the definitive answer; it is merely a guide. The correct homologue of a sample sequence cannot be definitely identified, until the whole genome has been sequenced.

### Conclusion

WuBLASTn appears to be reasonably good at identifying the coding sequences in close homologues at the DNA sequence level (assuming the results of Smith–Waterman to be correct) and more effective than ncbiBLASTn, when default parameters are used. The main difference being that WuBLASTn finds longer alignments. The ability of WuBLASTn and ncbiBLASTn to detect distant homologues was not the subject of this trial.

Table 5. Distribution and average alignment length for the 536 top hits found in common for all four sequence-search algorithms

| | wuBLASTn | ncbiBLASTn | Smith–Waterman | Fasta |
|---|---|---|---|---|
| Length of alignment (range) | 34–749 | 19–487 | 34–753 | 34–753 |
| Mean length | 331.5 | 215.0 | 335.5 | 329.8 |

Copyright © 2001 John Wiley & Sons, Ltd. 

### Acknowledgements

K.C.W. is supported by a BBSRC CASE award with AstraZeneca. Further support came from a grant to SGO from the Genes Development Committee of the BBSRC. Thanks go to Cary O'Donnell and Dyfed Lloyd Evans for their illuminating discussions, to Mathew Woodwark for his help and support, and to Mark Johnston for the sequence data.

### References

Altschul SF, Gish W, Miller W, Myers EM, Lipman DJ. 1990. Basic Local Alignment Search Tool. *J Mol Biol* **215**: 403–410.

Altschul SF, Madden TL, Schaffer AA, *et al.* 1997. Gapped BLAST and PSI-BLAST: A new generation of protein database search programmes. *Nucleic Acids Res* **25** (17): 3389–3402.

Fischer G, James SA, Roberts IN, Oliver SG, Louis EJ. 2000. Chromosomal translocations in the genome evolution of yeast are independent of speciation. *Nature* **405**: 451–454.

Goffeau A, Barrell BG, Bussey H, *et al.* 1996. Life with 6000 genes. *Science* **274**: 546–567.

KEGG ftpsite for *Saccharomyces cerevisiae* sequences: **ftp://kegg.genome.ad.jp/pub/genomes/sequences/S.cerevisiae**

Keogh RS, Seoighe C, Wolfe KH. 1998. Evolution of gene order and chromosome number in *Saccharomyces*, *Kluyveromyces* and related fungi. *Yeast* **14**: 443–457.

NCBI BLAST website: **http://www.ncbi.nlm.nih.gov/BLAST**

Naumov G. 1987. Genetic basis for classification and identification of the ascomycetous yeasts. *Stud Mycol* **30**: 469–475.

Pearson WR, Lipman DJ. 1988. Improved tools for biological sequence comparison. *Proc Natl Acad Sci U S A* **85**: 2444–2448.

Ryu S-L, Murooca Y, Kaneko Y. 1996. Genomic reorganisation between two sibling yeast species, *Saccharomyces bayanus* and *Saccharomyces cerevisiae*. *Curr Genet* **33**: 345–351.

Seoighe C, Wolfe KH. 1999. Updated map of duplicated regions in the yeast genome. *Gene* **238**: 253–261.

Smith TF, Waterman MS. 1981. Identification of common molecular subsequences. *J Mol Biol* **147**: 195–197.

SSearch available from the EBI website: **http://www.ebi.ac.uk/FTP/**

Washington University BLAST website: **http://blast.wustl.edu/**

Wolfe KH, Shields DC. 1997. Molecular evidence for an ancient duplication of the entire yeast genome. *Nature* **387**: 708–713.

*The*
**Genomics**
*Website at Wiley*

**www.wiley.co.uk/genomics**

The **Genomics** website at Wiley is a **DYNAMIC** resource for the genomics community, offering **FREE** special feature articles and new information **EACH MONTH**.

Find out more about *Comparative and Functional Genomics,* and *Proteomics*, and how to view many articles **FREE OF CHARGE**!

Visit the **Library** for hot books in Genomics, Bioinformatics, Molecular Genetics and more.

Click on **Journals** for information on all our up-to-the minute journals, including: *Genesis, Bioessays, Gene Function and Disease, Human Mutation, Genes, Chromosomes and Cancer* and the *Journal of Gene Medicine.*

Let the **Genomics** website at Wiley be your **guide** to genomics-related web sites, manufacturers and suppliers, and a calendar of conferences.

WILEY

2171

Copyright © 2001 John Wiley & Sons, Ltd. 
